# Retrograde sclerotherapy of male varicocele with veno-venous shunts – incidence and management: a single-centre experience

**DOI:** 10.1259/bjr.20221061

**Published:** 2023-03-03

**Authors:** Pietro Pitrone, Salvatore Silipigni, Alberto Stagno, Antonella Cinquegrani, Antonino Cattafi, Antonino Bottari

**Affiliations:** 1 Department of Biomedical and Dental Sciences and of Morphological and Functional Images, Section of Radiological Sciences, University of Messina, Messina, Italy; 2 Department of Imaging, Interventional Radiology Unit, University Hospital “G. Martino”, Messina, Italy

## Abstract

**Objectives:**

The aim of our study is to compare the incidence of veno-venous shunts in male varicocele and evaluate the possibility to exclude them with manual compression or/and scrotal ligation in order to carry out the procedure of retrograde sclero-embolization.

**Methods:**

In our retrospective study, all patients undergone retrograde sclerotherapy for varicocele in our Interventional Radiology Unit in the last four years were evaluated. Collaterals toward other venous shunts were identified and how many and which patients would be able to complete the procedure safely were considered.

**Results:**

Of the 91 patients, as many as 22 (*i.e.,* 24.17%) patients presented anatomical variants, consisting on shunting into left iliac vein (9 [9.89%]), lumbar left veins (3 [3.29%]), right iliac vein (1 [1.09%]), both iliac veins (1 [1.09%]), left femoral vein (1 [1.09%]) or a more proximal portion of the ISV itself without shunting (3 [3.29%]). Patients with duplication could benefit from a more distal injection in order to prevent back-flow; of the 19 left, nine successfully underwent sclerotherapy with manual compression or/and scrotal ligation, whereas in 10 flow through the collaterals could not be interrupted and patients were demanded for surgery.

**Conclusions:**

Many patients with abnormal communications between the internal spermatic vein and the iliac veins (that is, shunts towards the iliac veins) may as well undergo retrograde sclerotherapy safely if compression/ligation is applied.

**Advances in knowledge:**

No large previous study highlighted the impact of veno-venous shunts in technical feasibility of retrograde sclerotherapy of varicocele.

## Introductory section

Varicocele represents a common condition within the male population, arising at the adolescent age and becoming evident in the adult,^1,2^ representing the first cause of male infertility.^3–5^ Most cases occur on the left and are secondary to valvular lack or malfunctioning,^6^ although other minor causes (*e.g.,* the presence of aberrant communicating vessels) are advocated.^1^ The main indication for treatment is the presence of pain (at any age), infertility in adult patients,^7^ and testicular hypotrophy under 18 years of age (since a spermiogram is not obtainable in youngsters).^8,9^ From literature data, it is not shown as an absolute superiority between surgical and non-surgery or endovascular percutaneous treatment.^10^ Retrograde sclerotherapy represents the most available percutaneous approach, consisting in the catheterization of the internal spermatic vein (ISV) and injection of a sclerosing agent (SA) under fluoroscopy.^11^ Manual compression of the external inguinal ring or/and scrotal ligation is mandatory to avoid penetrance of the embolic agent into the pampiniform plexus, inducing inflammation and thrombosis, especially in those subjects with aberrant communicating veins from the ISV to iliac, lumbar or other venous collectors; Valsalva maneuver is mandatory as well to prevent renal reflux. Currently, no clear and univocal guidelines about the most appropriate management of patients with veno-venous shunts are available. The objective of our study is to assess the incidence of veno-venous shunts in male varicocele and evaluate the possibility of excluding them with manual compression or/and scrotal ligation, in order to perform retrograde sclero-embolization safely.

## Methods and materials/patients

We present a retrospective study on 95 patients (78 adults, 17 adolescents, mean age: 26.75 years, median age: 25 years, range: 12–54 years) subjected to percutaneous retrograde sclerotherapy of varicocele within the last four years in our Interventional Radiology Department. Clinical grading of varicocele before treatment (left in 84.2%, right in 3.1%, bilateral in 12.6%) was determined on color Doppler ultrasound according to Sarteschi classification (II in 6.3%, III in 72.6%, IV in 20 %, V in 1.0 %). Indications for treatment included testicular pain and discomfort, infertility, testicular atrophy, testicular or inguinal swelling, and prophylactic treatment. For patient under the age of 16–17, spermiogram was not obtained and indication to treatment were the presence of symptoms and a grade of varicocele superior to II according to Sarteschi classification. Patient’s characteristics and demographics date, imaging with venographic anatomy details, complications, and technical aspects were recorded in Table 1.

**Table 1.** Patients’ characteristics

Number of patients (n° tot): 95
Adult: 78
Adolescent: 17
Excluded: 4
Age: 12-54 Mean: 26.75 Median: 25
Grade (Sarteschi classification)
I: 0 % II: 6.3 % III: 72.6 % IV: 20.0 % V: 1.0 %
Affected side left: 80 (84.2 %) right: 3 (3.1 %) bilateral: 12 (12.6 %)
technical feasibility: 85.3% (81/95) technical infeasibility: 14.7% (14/95) technical success: 100% (81/81) clinical success after 1 month, 6 months and 1 year: 98.8% (80/81)

## Endovascular procedure

Percutaneous access was chosen based on patient comfort (right jugular, right femoral, or humeral). Ultrasound-guided percutaneous access was performed, under local anesthesia, with a 4 Fr sheath; with an angled hydrophilic guide wire (Radifocus; Terumo, Tokyo, Japan 0.035) and a 4-Fr catheter (Ber or Cobra C2) through the left renal vein, the internal spermatic vein was selectively catheterized under fluoroscopic guidance. Diagnostic phlebography was performed before and during Valsalva maneuver, in order to detect any potential collateral toward other venous collectors. If no contraindications were present, a mixture (foam) of 3% atoxysclerol 4 ml (ranging from 2 to 10 ml) and air (ratio 2:8) was injected and followed by a few mL of normal saline solution (to pull the remaining sclerosant out of the catheter lumen). Injection was performed during Valsalva maneuver with patient cooperation and manual compression of the external inguinal ring (for about two-three minutes), in order to extend action of the foam in the specific site and avoid non-target embolization. Varicocele anatomy was determined according to the classification by Bahren et al and the prevalence of each variant compared with prior studies in the literature. Patients were discharged the same day and a one-month, 6 months, and 1-year follow-up with Doppler ultrasonography was scheduled.

## Results

Of 95 patients, three patients were excluded from our analysis for a right-side disease with previous treatment of left varicocele; one more patient was excluded for varicocele and impaired renal drainage due to “nutcracker phenomena”.

From a final population consisting of 91 patients with left-side-primitive disease, 22 (*i.e.,* 24.17%) presented collateral vessels arising from the internal spermatic vein and shunting into the left iliac vein (9 [9.89%]), lumbar left veins (3 [3.29%]), right iliac vein (1 [1.09%]), both iliac veins (1 [1.09%]), left femoral vein (1 [1.09%]) or a more proximal portion of the ISV itself without shunting (3 [3.29%]).

**Figure 1.**
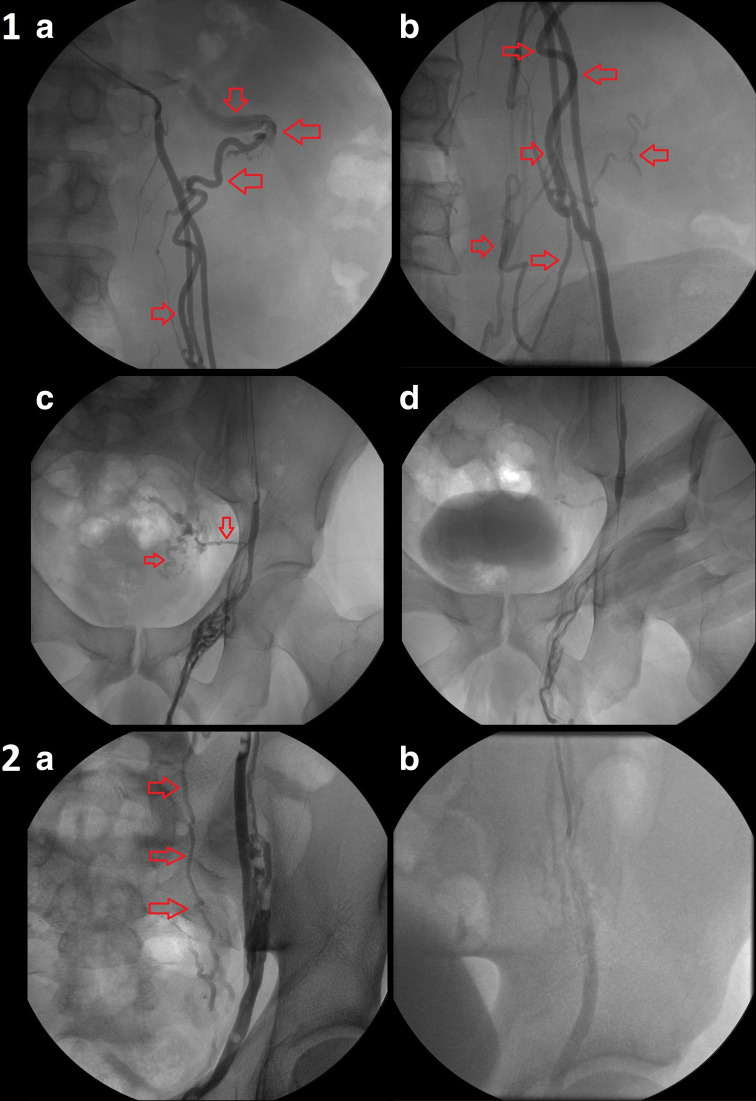
phlebographies from two different patients with duplication of the ISV (Bahren Class III). In patient 1, the ISV shows multiple duplications (red arrows), originating from the mean tract of the spermatic vein and directed to renal, retroperitoneal, and ureteral veins (fluoroscopy images at a higher level-A and B) to pelvic viscera through iliac vein branches (fluoroscopy images at a lower level-**C**); distal injection of the SA (at the level of the iliac branch) shows that collateral branches are not involved by reflux, while manual compression avoids penetration of the embolic agent into pampiniform plexus (**D**). In patient 2, additional retroperitoneal branches (red arrows) reach the level of the sacroiliac joint (**A**);distal injection (at the same level) allows the exclusion of these collaterals and technical success without complications.

**Figure 2.**
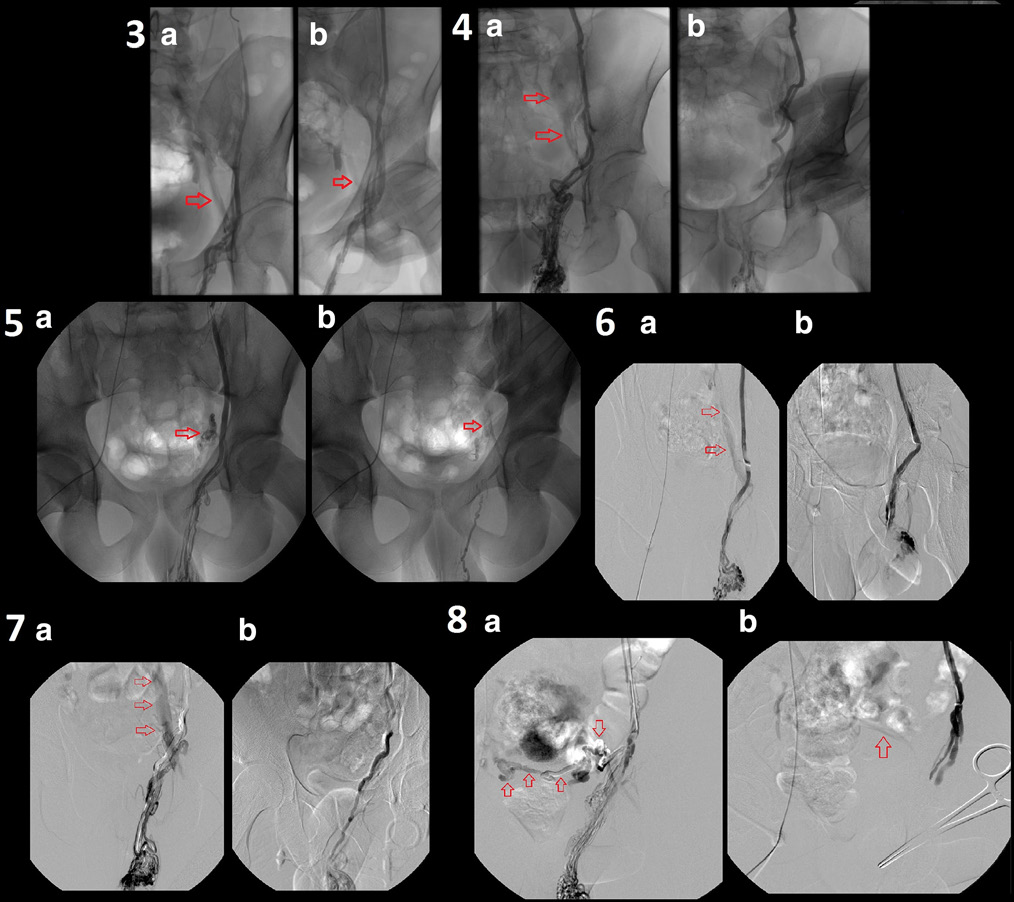
phlebographies from 6 patients (patients 3–**8**) successfully treated presenting flow through collaterals towards iliac veins (Bahren Class IV). Per each patient a homo-lateral iliac shunt is shown in figure A (red arrow) originating at the level of the internal inguinal ring and flow through shunts is excluded after manual compression at the level of the external inguinal ring or ileo-pubic branch; sometimes scrotal ligation (patients 6 and 7) was necessary to exclude shunting correctly. In patient eight contra lateral iliac shunt is seen at the level of the ileo-pubic branch (red arrows in A) and flow is reduced (red arrow in B) after scrotal ligation.

**Figure 3.**
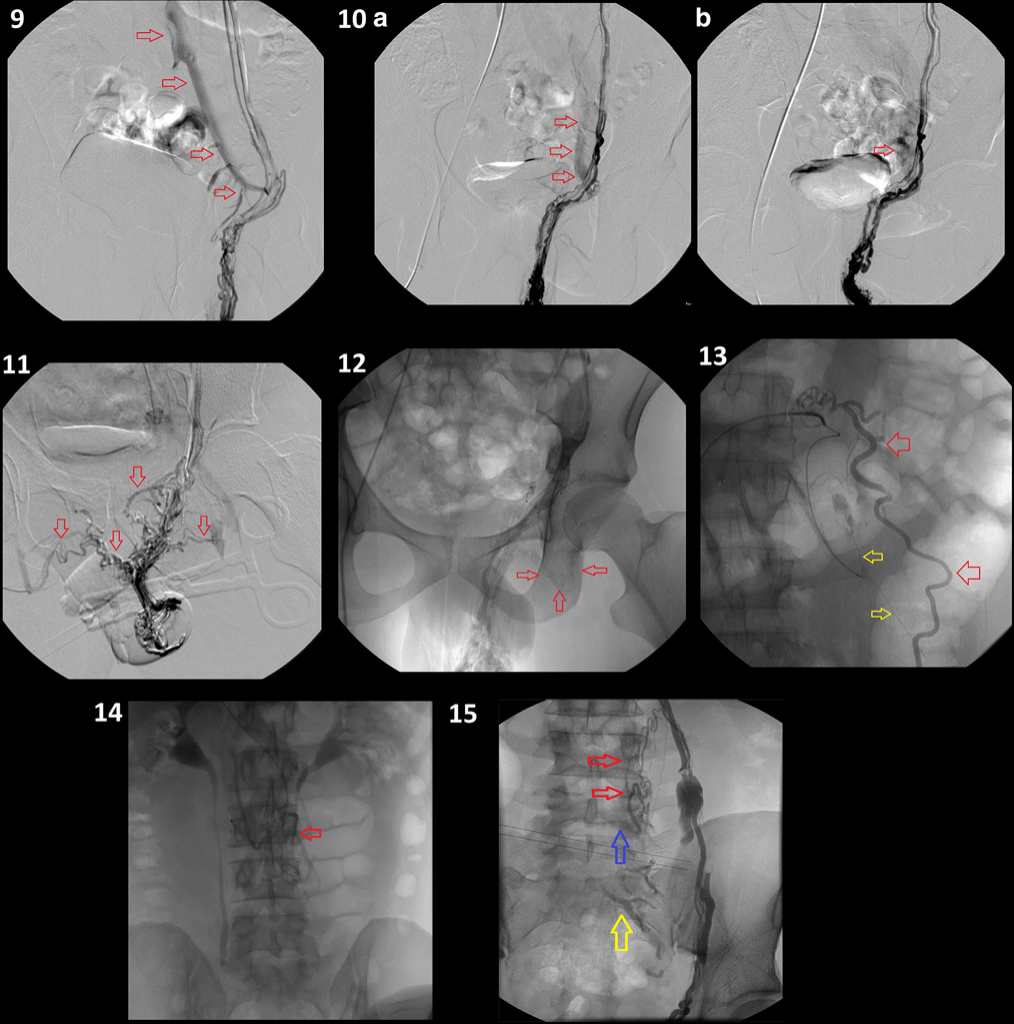
(patients 9–**15**): phlebographies from non-treated patients, where neither compression nor ligation yielded stop or adequate flow reduction through the collaterals (red arrows). Patients 9 and 10 DSA showed a massive left iliac shunt at the level of the internal inguinal ring (Bahren Class IV). Patient 11: DSA showing bilateral iliac shunts below the level of the coxofemoral joint, persisting after compression and ligation (Bahren Class IV). Patient 12: minimum left femoral shunt below the level of the coxofemoral joint (Bahren Class IV). Patient 13: a small-caliber vessel (yellow arrow) arising from the left renal vein anastomosing with the true ISV (red arrow), which drains into a lumbar collector (left paravertebral, level L2-L4, Bahren Class II). Patient 14: internal spermatic vein draining into a left lumbar collector (left paravertebral, level L3-L4; Bahren Class II). Patient 15: internal spermatic vein with collaterals towards pyelo-ureteral veins (level L3-L4; red arrows) draining into left iliac (Level S1; yellow arrow) and retroperitoneal veins (level L4; blue arrow; Bahren Class IV).

The incidence of collateral vessels and anatomic variants based on Bahren’s classification was established (Table 2) and a comparison with previous studies was made, with substantial similarity in some records (Table 3). Three patients with duplication without shunting (Bahren class III) only needed a more distal injection in order to prevent back-flow (Figure 1 **, patients 1–2**). Of the 19 left, in nine patients sclerotherapy proved feasible with successful treatment using manual compression or/and scrotal ligation (Figure 2 **, patients 3–8**); in the other 10 patients, the same maneuvers did not manage to stop flow through the collaterals (Figure 3 **, patients 9–14**), thus the procedure was interrupted and the patients were referred to surgical treatment. Notably, in subjects with shunts toward homolateral or/and controlateral iliac vein/veins, the above-mentioned maneuvers were often enough to stop or adequately reduce blood flow through the collaterals (9 out of 12 [75%]); this may be related to the possibility to perform a strong compression at the level of external inguinal ring (due to the low vessels-skin distance) or appropriate ligation (at the level of the scrotum, below the external inguinal ring). To the knowledge, within our cohort (22) no subjects from the treated subgroup (12) showed recurrence of homolateral varicocele at 1-month, 6-month, or 1-year follow-up ultrasound; in three patients a minimum reflus was reported during Valsalva maneuver.

**Table 2.** Venographic anatomy of left primary varicoceles (67)

Bahren anatomic type classification	Features	N° (%)
0	Single ISV with sufficient valves and no evidence of venous reflux	0 (0%)
I	Single ISV with insufficient or absent valves	69 (75.8%)
II	Reflux into a single ISV that communicates with collaterals to lumbar and/or retroperitoneal veins (≥2 ostia to renal vein)	3 (3.29%)
III	Reflux into a single incompetent ISV at renal vein junction, with caudal duplication	3 (3.29%)
IV	Reflux into ISV from segmental renal/retroperitoneal veins through valveless collaterals	16 (17.58%)
V	IVC or renal vein anomaly (bifurcation of the renal vein, circumaortic renal vein)	0 (0%)

ISV, internal spermatic vein.

**Table 3.** Prevalence (%) of the possible anatomical variations of the internal spermatic vein in our study compared with four different experiences.

Barhen’s class	Our experience (95)	Sigmund et al (717)^[^^12^^]^	Wunsch and Efinger (2047)^[^^13^^]^	Lenz et al (386)^[^^14^^]^	Sze et al (17)^[^^15^^]^
0	0	6.0	4.7	0	0
I	75.8	68.9	48.5	58.3	11.8
II	3.29	5.2	12.7	4.7	11.8
III	3.29	2.4	12.4	5.4	64.7
IV	17.58	15.9	18.9	27.2	5.9
V	0	1.7	2.8	4.4	5.9

## Discussion

By the term varicocele, we mean varicosity and tortuosity of the pampiniform plexus (PP), located in the spermatic cord around the testis, secondary to retrograde blood flow from the internal spermatic vein (ISV).^3^ The prevalence raises from pediatric (<1% under 10 years) to adolescent subjects (15%), supporting the progressive nature of the disease^1^; spontaneous regression is possible but infrequent.^16^ It is usually left-sided (78–93% of cases, versus 1–7% on the right and 2–20% bilateral)^17^ due to the perpendicular drainage of the ISV into the homolateral renal or suprarenal vein, together with an overall longer course and greater differences in pressure. However, an analogous fashion for the right ISV (usually draining into the inferior vena cava with an acute angle) is possible.^18^ Primary varicocele, due to valvular absence or insufficiency (found in 75% of venography studies)^6^ or aberrant communicating vessels draining into alternative venous collectors,^3^ must be distinguished from secondary forms caused by external compression, as in the so-called “nutcracker phenomenon”, where the left renal vein is squeezed between the aorta and the superior mesenteric artery.^19^ Other potential mechanisms include transient increases in arterial blood blow overcoming venous capacity of the PP,^20^ involution of collateral draining veins (“ontogenetic etiology”),^4^ a higher concentration of nitric oxide (vasodilator),^21^ altered motility of the fasciomuscular tube around ISV or atrophy of collagen within the wall of the spermatic cord.^22^ Most of the varicoceles are asymptomatic, with only scrotal or inguinal discomfort or pain, worsened after long periods of standing, with an incidence of 2–10% in adults, whereas adolescents are usually diagnosed incidentally.^23^ The main issue is actually infertility, reported in 5 to 20% of the male population and most evident in adult age.^2^ Reduced sperm count, motility, morphology, and alternative forms (a “stress pattern” with immature forms, amorphous cells, and tapered forms) are described^24,25^ as a result of testicular hypotrophy and altered spermatogenesis. Many mechanisms have been advocated, from increased scrotal temperature (due to stasis and reflux of warm blood)^26^ to excessive production of reactive oxygen species (ROS),^27^ chronic arteriolar vasoconstriction (due to incremented venous pressure, with subsequent hypoxia),^28^ high concentrations of adrenal cortical hormones (with arteriolar vasoconstriction)^29^ and FSH or low concentrations of testosterone (due to alterations in the hypothalamic-pituitary-gonadal axis)^30^ or inhibin B (correlated with testicular volume and Sertoli cell activity),^31^ toxic substances in the refluxing blood (*e.g.,* ROS and cadmium)^28,32^ and autoimmunity (antisperm antibodies due to the presence of breaches in the blood-testis barrier).^33^ Dilatation of the periprostatic venous plexus (DPVP) seems to reduce sperm motility and increase seminal fluid viscosity.^34^ Physical examination (sensitivity 50–70%)^4,35^ is followed by scrotal ultrasound (sensitivity 97%, specificity 94%)^36^: anechoic enlarged veins (>3 mm) with reflux lasting more than one second after Valsalva maneuver or showing spontaneously are present. Indications for treatment include discomfort or pain or male infertility in case of a clinically palpable varicocele, female regular fertility or correctable infertility, or one or more semen abnormalities^7^; correction actually improves both sperm count and motility.^37,38^ When dealing with adolescents, since fertility is not well predictable yet, one must consider presence of the variable symptomatic expressions of disease and the presence of testicular hypotrophy (difference in a testicular volume greater than 2 ml or 20%) and, of lesser importance, abnormal semen parameters (when available).^8,9^ When considering the rare right varicocele, another indication is represented by a persistent or recurrent left varicocele in the absence of evident collaterals.^16^

The options available for treatment of varicocele are surgery (ligation of the veins within the spermatic cord at the inguinal canal or the subinguinal area) or endovascular scleroembolization (antegrade or retrograde sclerotherapy).^4^ Retrograde sclerotherapy represents nowadays the most used, effective, and safe percutaneous technique in the treatment of male varicocele and a valid alternative to surgery.^39^ Via a common femoral, internal jugular, or humeral venous access, the spermatic vein is catheterized and its distal portion (lower edge of the ischiopubic ramus) is injected with a sclerosing agent (SA). The patient is asked to perform Valsalva (or pressure on his hypochondrium is applied) in order to avoid the reflux of the SA and a distal barrage near the external inguinal ring (that is, the highest level of the scrotum) is placed to prevent the scrotal veins from phlebitis. When dealing with large SV or patients with bidirectional flow due to augmented cardiac output, a temporary (about 10 min) proximal occluding balloon (OB, 4–8 mm, through which the SA is injected from the upper margin of the iliac bone) can replace Valsalva maneuver, favoring prolonged contact of the SA with the vessel wall, not influenced by vessel caliber or flow or the patient’s ability to keep Valsalva (especially in subjects with pain or needing sedation) and a reduced risk of venous rupture.^11,40^ We employed a mixture of 2–6 ml aetoxisclerol (polidocanol -a local anesthetic acting as a detergent SA, mostly used in Europe-) mixed with air to form a foam which may easily distribute throughout ISV and potential collaterals. Sodium tetradecyl sulfate (STS) and, less likely, hydroxy-polyethoxy-docanol and sodium morrhuate, represent valid alternatives to aetoxisclerol.^41–43^ In case of persisting reflux at post-sclerotherapy phlebography coils or glue should be used. Embolization through these alternative techniques is widely validated but possible complications must be well known by the operator: coil may migrate or cause venous dissection or perforation and detectable coils are preferred^44^; cyanoacrylate glue may also be employed in case of post-surgical recurrences but frequent complications are fixation of the delivery catheter, migration into the pulmonary circulation or phlebitis.^45^ However, some Operators even use primarily glue and, to a lesser extent, coils or/and vascular plugs (up- and downstream [“sandwich technique”] in order to prevent both thrombophlebitis of the pampiniform plexus and reflux) for the lower recurrence rate.^46^ No significant difference in terms of pain/discomfort or complications has been reported, although glue grants lower costs, radiation exposure, and recurrence rates, being effective also in the presence of collateral vessels and without risks of migration or need for large sheaths.^47^ Again, no substantial difference was observed using different types of mechanical agents like pushable hydrogel-coated and non-coated platinum and detachable fibered coils.^48^ Sclerotherapy’s advantages over surgery are the lower risks of hydrocele^49^ and testicular atrophy (in 3–33 and 1% of varicocelectomies), associated with injuries of lymphatic and arterial vessels, respectively^50,51^; its main risks are venous thromboembolism, thrombophlebitis and allergic reactions.^52^

Postoperative pain at 24–48 h may occur secondary to the inflammatory reaction associated with the SA (acting for 45–95 days)^53^ and conservative therapy (NSAD and cryotherapy) usually suffices.^16^ To date, there is no evidence demonstrating the absolute superiority of one technique over the other for varicocele treatment,^10^ since technical success is around 100% in both. When undergoing sclerotherapy, however, the patient remains in observation for 4 h only, and the only recommendation is not to perform any strong effort for the first 10–15 days.^16^

As previously described, varicocele might be caused by collateral veins draining into the iliac (3–5%), lumbar (3%), distal or capsular renal (5%), colonic vessels, or the contralateral scrotum (rarely)^54^; in general, duplications are described in 74% of cases.^55^ In particular, Bahren’s venographic classification distinguishes five types, depending on the absence of reflux (0), reflux into a single gonadal vein (I), a single gonadal vein communicating with accessory gonadal, lumbar, iliac vein or vena cava (II), a gonadal vein duplicated caudally but with a single trunk for the renal vein (III), a renal hilar/capsular vein communicating with the gonadal vein (IV) and a gonadal vein draining into a circumaortic renal vein (V).^54^ We have already reported the distribution of our patients within these categories; as previously noticed, no substantial differences are seen when compared with other studies from the literature, even considering the high variability of the number of patients included in each cohort.^12–15^ The presence of these aberrant communications may contraindicate sclerotherapy due to the risk of SA reflux, with these patients being often demanded to surgery.

## Conclusion

The presence of abnormal communicating vessels from the internal spermatic vein into other venous collectors represents a relative contraindication to retrograde sclerotherapy, due to the risk of reflux of the sclerosing agent. Manual compression or/and scrotal ligation can often reduce or stop blood flow through these collaterals. Despite the small cohort of patients included in our study (which explains the partial correspondence with data from the literature), we demonstrated how shunts towards the iliac veins are the most susceptible to our maneuvers, probably due to the proximity of the anatomical region and the possibility for ligation. Thus, most of these patients may safely complete the procedure, without requiring surgery. The main limitation of the study was the small cohort of patients involved (95 with only 22 presenting with anatomical variants) in comparison with other cohorts (Table 3); thus, larger studies should be carried out in order to support Our findings and suggest appropriate guidelines when dealing with veno-venous shunts.
